# Exopolysaccharide Produced by Probiotic *Bacillus albus* DM-15 Isolated From Ayurvedic Fermented *Dasamoolarishta*: Characterization, Antioxidant, and Anticancer Activities

**DOI:** 10.3389/fmicb.2022.832109

**Published:** 2022-03-03

**Authors:** Annadurai Vinothkanna, Ganesan Sathiyanarayanan, Amit Kumar Rai, Krishnamurthy Mathivanan, Kandasamy Saravanan, Kumaresan Sudharsan, Palanisamy Kalimuthu, Yongkun Ma, Soundarapandian Sekar

**Affiliations:** ^1^School of Food and Biological Engineering, Jiangsu University, Zhenjiang, China; ^2^Department of Biotechnology, Bharathidasan University, Tiruchirappalli, India; ^3^Institute of Chemistry, University of Neuchâtel, Neuchâtel, Switzerland; ^4^Institute of Bioresources and Sustainable Development, Regional Centre, Gangtok, India; ^5^School of Minerals Processing and Bioengineering, Central South University, Hunan, China; ^6^Department of Biochemistry, Bharathidasan University, Tiruchirappalli, India; ^7^Department of Chemistry, The Gandhigram Rural Institute (Deemed to be University), Dindigul, India

**Keywords:** probiotics, *Bacillus ablus*, exopolysaccharides, characterization, antioxidant, anticancer potential

## Abstract

An exopolysaccharide (EPS) was purified from the probiotic bacterium *Bacillus albus* DM-15, isolated from the Indian Ayurvedic traditional medicine *Dasamoolarishta*. Gas chromatography-mass spectrophotometry and nuclear magnetic resonance (NMR) analyses revealed the heteropolymeric nature of the purified EPS with monosaccharide units of glucose, galactose, xylose, and rhamnose. Size-exclusion chromatography had shown the molecular weight of the purified EPS as around 240 kDa. X-ray powder diffraction analysis confirmed the non-crystalline amorphous nature of the EPS. Furthermore, the purified EPS showed the maximum flocculation activity (72.80%) with kaolin clay and emulsification activity (67.04%) with xylene. In addition, the EPS exhibits significant antioxidant activities on DPPH (58.17 ± 0.054%), ABTS (70.47 ± 0.854%) and nitric oxide (58.92 ± 0.744%) radicals in a concentration-dependent way. Moreover, the EPS showed promising cytotoxic activity (20 ± 0.97 μg mL^–1^) against the lung carcinoma cells (A549), and subsequent cellular staining revealed apoptotic necrotic characters in damaged A549 cells. The EPS purified from the probiotic strain *B. albus* DM-15 can be further studied and exploited as a potential carbohydrate polymer in food, cosmetic, pharmaceutical, and biomedical applications.

## Introduction

Exopolysaccharides (EPSs) are structurally heterogeneous carbohydrate polymers produced by microorganisms (bacteria, yeast, fungi, and microalgae). Microorganisms produce EPSs in the presence of excess carbon sources or under extreme environmental conditions as a firmly associated cell-bound capsule or a loosely attached slime layer (Lynch et al., 2018; Andrew and Jayaraman, 2020). Bacterial EPSs are functionally involved in biofilm formation and environmental stress management to protect the bacterial cells from harsh conditions. EPSs also act as potential antigenic determinants in certain Gram-negative bacteria, i.e., *Klebsiella pneumoniae* capsular acidic polysaccharides (K antigen), which provide solid support to the bacteria to combat the host immune systems (Ma et al., 2018; Andrew and Jayaraman, 2020). Bacterial EPSs are widely used in pharmaceutical, food, petroleum, and cosmetic industries as emulsions, thickening agents, surfactants, viscosifiers, flocculants, additives, and preservatives (Freitas et al., 2011; Lynch et al., 2018). In addition, some bacterial EPSs also possesses anti-inflammatory, antitumor, antioxidants, antibacterial, antiviral, cholesterol-lowering, prebiotic, and immunomodulatory activities (Freitas et al., 2011; Saadat et al., 2019; Park et al., 2022). At present, EPSs are commercially available in the market as xanthan, gellan, dextran, alginates, curdlan, acetan, succinoglycan, and hyaluronan, and these polymers are generally non-toxic to the environment, biocompatible, and completely biodegradable (Freitas et al., 2011; Andrew and Jayaraman, 2020; Daba et al., 2021).

The Indian traditional fermented medicines have treated various diseases for many centuries. Recent studies have demonstrated the diverse microbial communities, especially probiotic bacteria from the traditional fermented foods and polyherbal fermented traditional medicines, namely *Arishta* (fermented decoctions) and *Asava* (fermented infusions). Polyherbal formulations’ composition and medicinal properties were extensively studied, and the fermentation processes were mainly mediated by self-generated microbial communities (Saadat et al., 2019; Sekar and Vinothkanna, 2019; Vinothkanna and Sekar, 2019). Different EPSs were purified from diverse bacteria, including probiotic bacterial strains isolated from traditional fermented foods and beverages (Hu et al., 2019; You et al., 2020; Park et al., 2022). Our previous study also showed a novel EPS polymer from the *Bacillus licheniformis* AG-06 isolated from the Indian Ayurvedic polyherbal fermented traditional medicine (Vinothkanna et al., 2021). Hence, probiotic microorganisms from the Indian Ayurvedic polyherbal formulations can be further exploited to produce food-grade EPSs with medicinal properties. EPSs from probiotic bacteria had shown significant rheological, flocculation, emulsification, and pharmaceutical properties (i.e., anticancer and immunomodulation) (Insulkar et al., 2018; Ramamoorthy et al., 2018; Vinothkanna et al., 2021). However, EPSs purified from probiotic bacteria isolated from traditional fermented medicines are mostly unknown, and assessing their therapeutic potentials can lead to an exciting opportunity for nutraceutical applications.

Functional groups present in the EPSs are mainly responsible for many biological properties. For example, sulfate groups in the bacterial EPS are considered potential lead for the antiproliferative, anticoagulant, fibrinolytic, antimicrobial, prebiotic, and wound-healing properties (Haroun-Bouhedja et al., 2000; Ruiz-Ruiz et al., 2011; Almutairi and Helal, 2021). Sulfated exopolysaccharide purified from the *Anoxybacillus gonensis* YK25 showed significant anticancer activity against the lung cancer cells (Karadayi et al., 2021). In addition, sulfated EPS (levan) derived from the *Bacillus megaterium* PFY-147 had shown free radical scavenging activity, antioxidant and probiotic activities, which validate the potential implications of sulfated EPS in biomedical applications (Pei et al., 2020). Therefore, exploring the sulfated EPSs from the probiotic bacteria isolated from the fermented medicine is essential to understand the role of EPSs in fermented medicines and their therapeutic interventions.

To meet all these challenges, we have systematically studied the EPS derived from the probiotic bacterium *Bacillus albus* DM-15 isolated from polyherbal fermented traditional medicine (*Dasamoolarishta*) of Indian Ayurveda. The purified EPS was characterized by various analytical methods, including two-dimensional nuclear magnetic resonance (NMR) spectroscopic analysis. Functional properties such as flocculation, emulsification and *in vitro* biological activities, including antioxidant and anticancer potential, were determined.

## Materials and Methods

### Chemicals and Reagents

Trifluoroacetic acid (TFA), monosaccharide standards (glucose, galactose, rhamnose, mannose, xylose, and arabinose), deuterium oxide (D_2_O), L-ascorbic acid, 2-diphenyl-1-picrylhydrazyl (DPPH), 2, 2’-azino-bis 3-ethylbenzothiazoline-6-sulfonic acid (ABTS), 3-(4,5-dimethylthiazol-2-yl)-2,5-diphenyl-2H-tetrazolium bromide (MTT), Acridine orange/ethidium bromide (AO/EB) and 4’,6-diamidino-2-phenylindole (DAPI) were procured from Sigma-Aldrich (Bangalore, India). Further, sodium nitroprusside, benzene, n-Hexane, n-Hexadecane, toluene, and xylene were purchased from Sisco Research Laboratories (Mumbai, India). Kaolin clay, Nutrient agar, DMEM (Dulbecco’s Modified Eagle’s Medium), FBS (Fetal Bovine Serum) and absolute alcohol were obtained from HiMedia (Mumbai, India). All chemicals and reagents used were of analytical and ultrapure grade.

### Bacterial Strain and Culture Conditions

The probiotic bacterial strain, DM-15, was isolated from the polyherbal fermented traditional medicine (*Dasamoolarishta*) of Indian Ayurveda. Strain DM-15 can synthesize EPS on ruthenium red milk agar (Vinothkanna and Sekar, 2019) and it was identified by biochemical tests and molecular characterization by 16S rRNA gene sequencing (Vinothkanna and Sekar, 2019). The 16S rRNA sequences were submitted to GenBank with an accession number of MW031905, and the phylogenetic analysis was performed by the neighbor-joining method using MEGA software (version 7.0). Strain DM-15 was routinely cultured on nutrient agar (NA) plates, and the seed culture was prepared in lysogeny broth and incubated at 30°C for 24 h. The EPS production was carried out in 500 mL of Erlenmeyer flask containing 100 mL of lysogeny broth medium amended with 2% sucrose. About 2% seed culture (∼10^6^ cells mL^–1^) was inoculated into the production medium and incubated at 30°C for 5 days (Vinothkanna et al., 2021).

### Extraction and Purification of Exopolysaccharide From Strain DM-15

After the incubation, the bacterial fermented broth was centrifuged at 8,000 × g, and cell-free supernatant (CFS) was collected. The pooled CFS was thermally treated at 80°C for 20 min to deactivate the carbohydrate polymer degrading enzymes. The CFS was concentrated in a rotary evaporator, and two volumes of absolute ice-cold ethanol (99%) were added to the CFS and kept at 4°C for 24 h. The crude EPS precipitate was then recovered by centrifugation at 12,000 × g for 20 min at 4°C, and the precipitate was dissolved in deionized water. Next, the crude EPS was dialyzed against sterile deionized water for 12 h at 4°C. In addition, EPS was purified using ion-exchange DEAE-Sepharose fast flow column (1.6 × 25cm) with sodium chloride (0–0.7 M) gradient as the eluent at a flow rate of 36mL/h by column chromatography. Gel-filtration chromatography was performed using a Sepharose-4B column (1.6 × 95cm) with deionized water as the eluent at a flow rate of 12mL/h (Sun et al., 2015). The eluted fractions were periodically checked for the carbohydrate content by the phenol-sulfuric acid assay. Following purification, the EPS fractions were pooled, lyophilized, and used for further characterization.

### Characterization of the Purified Exopolysaccharide

#### Chemical Constituents Analysis

About 100 mg of lyophilized EPS was dissolved in deionized water, and the total carbohydrate was analyzed by a phenol sulfuric acid method where glucose was used as a standard (Dubois et al., 1956). Total protein was determined by Bradford assay with bovine serum albumin (BSA) as a standard (Bradford, 1976). The sulfate content was estimated using K_2_SO_4_ as a standard according to the method described in previous literature (Terho and Hartiala, 1971).

#### Monosaccharide Composition and Molecular Weight Analysis

Briefly, 2 M TFA was used to hydrolyze 5 mg of purified EPS at 120°C for 2 h. For monosaccharide composition analysis, about 10 μL of hydrolyzed EPS was injected into GC-MS (QP-2010; Shimadzu Corporation, Japan), equipped with Rtx column (60 m × 0.25 mm I.D × 0.25 μm thickness (Restek, United States). The chromatographic conditions used were as follows: Helium was used as a carrier gas (99.99% purity) with a flow rate of 10 mL min^–1^. The oven temperature was initially set at 150°C for 1 min, then elevated to 300°C at a rate of 8°C/min, and subsequently increased to 320°C at a rate of 7°C/min for 10 min. The ion source and interface temperatures were set at 230°C and 300°C, respectively. The mass ion (m/z) range was 50–500. The retention time (RT) of the monosaccharides present in the purified EPS was compared to that of monosaccharide standards (glucose, galactose, rhamnose, mannose, xylose, and arabinose) (Ayyash et al., 2020).

The molecular weight of the EPS was determined by size-exclusion chromatography equipped with a TSK Gel G5000PW column (7.5 × 300 mm, Tosoh Biosciences). The column was equilibrated with 50 mM ammonium acetate buffer (pH 5.5) and calibrated. Dextran standards (1,189, 759, 511, and 167 kDa) and glucose, 50 μL EPS solution (2 mg/ml) were separately loaded onto the column and were eluted with 50 mM ammonium acetate buffer (pH 5.5) at 1 mL min^–1^ (Sun et al., 2015). An evaporative light scattering detector was used to detect soluble components eluted from the column, and data were collected and processed by Agilent ChemStation software (Agilent Technologies, United States).

#### Spectral Analyses

About 1 mg mL^–1^ of purified EPS was dissolved in deionized water, and the absorbance spectra were measured at a range of 190–800 nm using a UV-VIS spectrophotometer (Jasco V-650, Tokyo, Japan) (Zhou et al., 2016). The lyophilized EPS (10 mg) was homogenized with potassium bromide (KBr) and pressed into a pellet (1 mm thickness, 10 mm diameter). Fourier-transform infrared (FT-IR) (PerkinElmer, Spectrum Two, United States) spectrum was recorded from 4,000 to 400 cm^–1^ for the detection of major functional groups present in the purified EPS (Vinothkanna et al., 2021). In addition, the crystalline quality, phase composition, purity, and crystal structure of the EPS were characterized by an X-ray diffractometer (D/MAX Ultima III, Rigaku Corporation, Tokyo, Japan) with Cu-Kα radiation (1.54056 Å) over a 2θ Scan range of 10°–80°. The step length and step time used were 0.01° and 0.1 s/step, respectively (Mathivanan et al., 2021).

Briefly, 30 mg of EPS was partially hydrolyzed with 0.1 M TFA at 100°C for 60 min. The partially hydrolyzed EPS was further dialyzed against deionized water and lyophilized (Noseda et al., 2020). Finally, the partially hydrolyzed EPS was dissolved in 99.99% deuterium oxide (D_2_O). ^1^H NMR (5 mg mL^–1^), ^13^C NMR (40 mg mL^–1^), and two-dimensional (2D) NMR analyses such as ^1^H-^1^H correlated spectroscopy (COSY) and ^13^C-^1^H heteronuclear single quantum coherence (HSQC) were recorded at 400 MHz, Bruker Avance spectroscopy (Bruker Co., Billerica, MA), respectively (You et al., 2020).

#### Microscopic Analysis

The topographical image of purified EPS was analyzed by atomic force microscopy (AFM; Agilent 5500 model, United States). About 1 mg mL^–1^ of purified EPS was suspended in deionized water and kept in a water bath at 50°C under sonication. The completely dissolved EPS was chilled to room temperature and diluted to 10 μg mL^–1^ concentration. About 5 μL of EPS aqueous solution was fixed on a fresh silicon material by spin-coating method and desiccated overnight and studied using AFM with non-contact-mode (Vinothkanna et al., 2021). The surface microstructure of the purified EPS was analyzed using a field emission scanning electron microscope (FE-SEM; Carl Zeiss—Sigma model, Germany). The purified EPS was mounted onto an aluminum metal stub and gold-sputtered with 10 nm thickness. The FE-SEM micrographs were obtained at an accelerating voltage of 20 kV (Li et al., 2015).

### Flocculation Activity of the Exopolysaccharide

About 1 mL of EPS (5–50 mg L^–1^) was mixed with 8 mL kaolin suspension (5 g L^–1^) and 1 mL of calcium chloride solution (1%) in a test tube (Sathiyanarayanan et al., 2014) and vortexed well and left to stand for 5 min. The absorbance of the upper layer was measured at 550 nm using a UV-VIS spectrophotometer (Jasco V-650, Tokyo, Japan), wherein sterile distilled water was used as a control. Flocculation activity (%) of the purified EPS was calculated as follows:


(1) f=eControl-Sample/Control×100

### Emulsification Property of the Exopolysaccharide

About 2 mL of the purified EPS (0.025, 0.5, and 1%) was mixed with polar and non-polar solvents (Benzene, n-Hexane, n-Hexadecane, Toluene, and Xylene) at a 1:1 ratio (v/v). The mixture was vortexed well and left to stand at room temperature for 24 h, and the emulsifying index (*E*_*h*_) was calculated as follows:


(2) E=h(H/ELH)S×100

where *H*_*EL*_ is the emulsion layer height, and HS is the solution mixture’s total height (Abid et al., 2021).

### Antioxidant Potential of the Exopolysaccharide

The antioxidant activity of the purified EPS (0.5–3.0 mg mL^–1^) was evaluated by different methods such as DPPH, ABTS, and nitric oxide (NO) radical scavenging test (Ma et al., 2018). In addition, the absorbance of the reaction mixture was measured by UV-VIS spectrophotometer (JASCO, Model V-650, Japan) at 517 nm (0.2 M DPPH), 734 nm (7 mM ABTs), and 546 nm (5 mM Sodium nitroprusside), respectively. L-Ascorbic acid (0.5–3.0 mg mL^–1^) and deionized water were used as the positive and negative controls, respectively (Ma et al., 2018; Sivasankar et al., 2018; Vinothkanna et al., 2021).

### Anticancer Potential of the Purified Exopolysaccharide

#### Cytotoxicity Assay

Cytotoxic activity of the EPS was tested against the human lung cancer cell line (A549) by MTT assay. Briefly, A549 cells were cultured in 96 well plates (flat bottom) containing the mixture of DMEM, FBS (10%), penicillin (100 U mL^–1^), and streptomycin (100 mg L^–1^) under the humidified environment (37°C, 5% CO_2_) for 24 h. Various concentrations (10–100 μg mL^–1^) of the purified EPS (200 μL) was mixed with A549 cells at a concentration of 5 × 10^3^ cells/well and left to stand for 24 h at 37°C. Then 20 μL of MTT reagent (5 mg mL^–1^ in phosphate-buffered saline) was added to the reaction wells and allowed to react for 4 h (Vinothkanna et al., 2021). Further, the purple formazan crystals were dissolved by adding 100 μL of deionized water. The reduction of MTT-formazan was determined at 570 nm using a microplate reader (Bio-Rad, iMark, United States). Cellular inhibitory effect (%) was determined using the following formula:


(3) Cellviability(%)=A-ControlA/TreatedcellsA×Control100

#### Acridine Orange/Ethidium Bromide and 4′,6-Diamidino-2-Phenylindole Staining

The purified EPS (IC_50_ dose) and A549 tumor cells were mixed and incubated for 24 h 37°C under a 5% CO_2_ environment. The treated cells were harvested and washed with phosphate-buffered saline. About 25 μL of AO/EB (Kasibhatla et al., 2006) and DAPI (Wang et al., 2019) stains were mixed with the EPS-treated cancer cells on a glass slide and observed under a fluorescence microscope (Carl Zeiss, Axioscope2plus).

### Statistical Analysis

All experiments were performed in triplicate, and the data were represented as mean ± standard deviation. The data of antioxidant assays were compared by two-way ANOVA using Dunnet’s multiple comparison tests, and *p < 0.05, ^**^p < 0.01, and ^***^p < 0.001 were considered statistically significant.

## Results and Discussion

### Production of Exopolysaccharide

**FIGURE 1:**
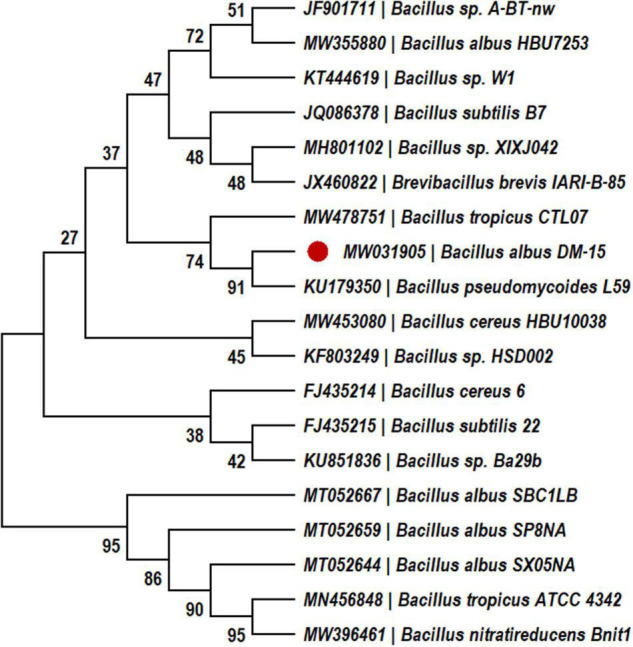
Phylogenetic tree constructed using MEGA 7.0. by neighbor Joining method.

In our previous study, probiotic strain DM-15 was isolated from the Indian Ayurvedic medicine *Dasamoolarishta*, and its EPS producing ability was identified (Vinothkanna and Sekar, 2019). The strain DM-15 had shown 100% similarity with *Bacillus albus* reference strains during the 16S rRNA phylogenetic analysis (Figure 1), and biochemical tests also validate the *Bacillus* genus. Therefore, based on the biochemical and phylogenetic analysis, probiotic strain DM-15 isolated from the fermented medicine was designated as *B. albus* DM-15. Furthermore, the EPS production experiment showed about 290 ± 0.78 mg L^–1^ of EPS from *B. albus* DM-15 after a week of incubation. This production was comparatively higher than the previously reported *Bacillus* spp. such as *Bacillus subtilis* MKU SERB2 (147.23 mg L^–1^) and *Bacillus* sp. S-1 (35 mg L^–1^) isolated from fermented Sichuan pickles (Hu et al., 2019; Sathishkumar et al., 2021). The EPSs synthesis is a natural phenomenon in probiotic bacteria, and it plays a vital role in biofilm formation, bacterial adhesion, bacterial cell aggregation, water-holding ability, attracting the nutrient sources, and protective barrier (Nwodo et al., 2012).

### Chemical Constituents, Monosaccharide Profile, and Molecular Weight of the Exopolysaccharide

Ion-exchange chromatography had shown two different EPS peaks from the DEAE-Sepharose column (Supplementary Figure 1). Among these, the initial fraction was too sparse to harvest; however, a substantial proportion of the subsequent fraction was appropriately collected and further subjected for purification by gel-filtration chromatography using a Sepharose 4B column (Supplementary Figure 2). The single fraction was eluted from the gel-filtration chromatography and harvested and analyzed by a UV-visible spectrophotometer. UV-visible spectra indicated a band at 195 nm, and no other absorbance peaks were detected between 260 and 290 nm (Supplementary Figure 3), which validates the purity of the EPS (Zhou et al., 2016). Size-exclusion chromatography indicated that the molecular weight of the purified EPS was approximately 240 kDa. The total carbohydrate, protein, and sulfate content were 852.78, 27.51, and 119.71 mg g^–1^. Carbohydrate contents of the EPSs were higher than protein and sulfated content, a typical feature that was observed with other bacterial EPSs (Sathiyanarayanan et al., 2014, 2016). The monosaccharide composition analysis had shown the presence of glucose, galactose, xylose and rhamnose in the GC-MS chromatogram (Supplementary Figure 4), which confirms the heteropolymeric nature of the EPS purified from *B. albus* DM-15. The peak area, retention time, and mass ratio were summarized in Supplementary Table 1. Glucose (71.32%) had shown a higher molar percentage followed by galactose (13.55%), xylose (9.38%), and rhamnose (5.75%), respectively. Similar monosaccharide composition was identified from *B. tequilensis* FR9M76, *B. licheniformis* PASS26, and *B. licheniformis* BL-P1, where five or six monosaccharides composed the polymeric structure of the EPS (Rani et al., 2017; Insulkar et al., 2018; Xu et al., 2019). The monosaccharide composition may vary from strain to strain in the same species due to the influences of nutrients in the growth medium and other external variables such as pH, water activity, temperature, etc. (Angelin and Kavitha, 2020).

### Structural Properties of the Exopolysaccharide

#### Fourier-Transform Infrared Analysis

**FIGURE 2:**
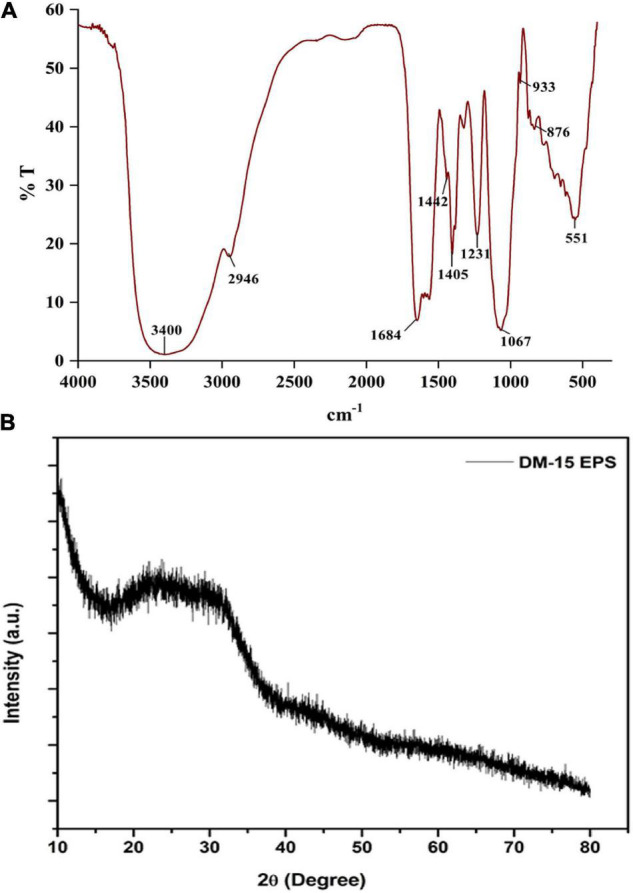
**(A)** FT-IR spectrum and **(B)** XRD pattern of the EPS purified from *B. albus* DM-15.

The FT-IR spectra revealed distinct polysaccharide functional groups at 3,400, 2,946, 1,684, 1,405, 1,231, and 1,067 cm^–1^ (Ramamoorthy et al., 2018). A strongly stretched band at 3,400 cm^–1^ indicated the hydroxyl functional group (–OH) (Figure 2A). The band stretched at 2,946 cm^–1^ corresponds to the bending vibrations of the C-H group (Sathiyanarayanan et al., 2016; Park et al., 2022). An intense peak recorded at 1,684 cm^–1^ related to the carbonyl (*C* = O) bending vibration (Sardari et al., 2017; Park et al., 2022). The absorption band at 1,405 cm^–1^ indicates the characteristics of the COO group. The peaks at 1,231 and 1,067 cm^–1^ might be the pyranose ring (Vijayabaskar et al., 2011). A solid peak at 933 cm^–1^ due to the presence of glycosyl residue, particularly by the β-pyranose configuration in EPS. The weak band at 876 cm^–1^ corresponds to the pyranose ring’s of the monosaccharides (Li et al., 2015; Saravanakumar et al., 2021). The absorption signal at 834 cm^–1^ is assigned to C = O = S elastic vibration. A short band at 618 cm^–1^ indicates the adjustable pulse of alkynes and an intense peak at 551 cm^–1^ typical signature of alkynes and aldehydes. Overall, the FTIR spectrum authenticates the carbohydrate polymeric functional groups, playing a significant role in the functional and biological activities of the EPS (Sathiyanarayanan et al., 2016).

#### X-Ray Diffraction Analysis

The diffractogram demonstrated a significant broad non-symmetric signal found at 23.8° (2θ), which indicates the amorphous (non-crystalline) nature of the EPS (Figure 2B). Similar non-crystalline EPS have been identified from *L. fusiformis* KMNTT-10 and *B. cereus* KMS3-1 (Krishnamurthy et al., 2020; Mathivanan et al., 2021). Amorphous natural biopolymers can be used as thickening agents in the food processing industries apart from their application to produce edible films and coatings (Kumar Bajaj et al., 2015).

#### Nuclear Magnetic Resonance Spectroscopic Analysis

**FIGURE 3:**
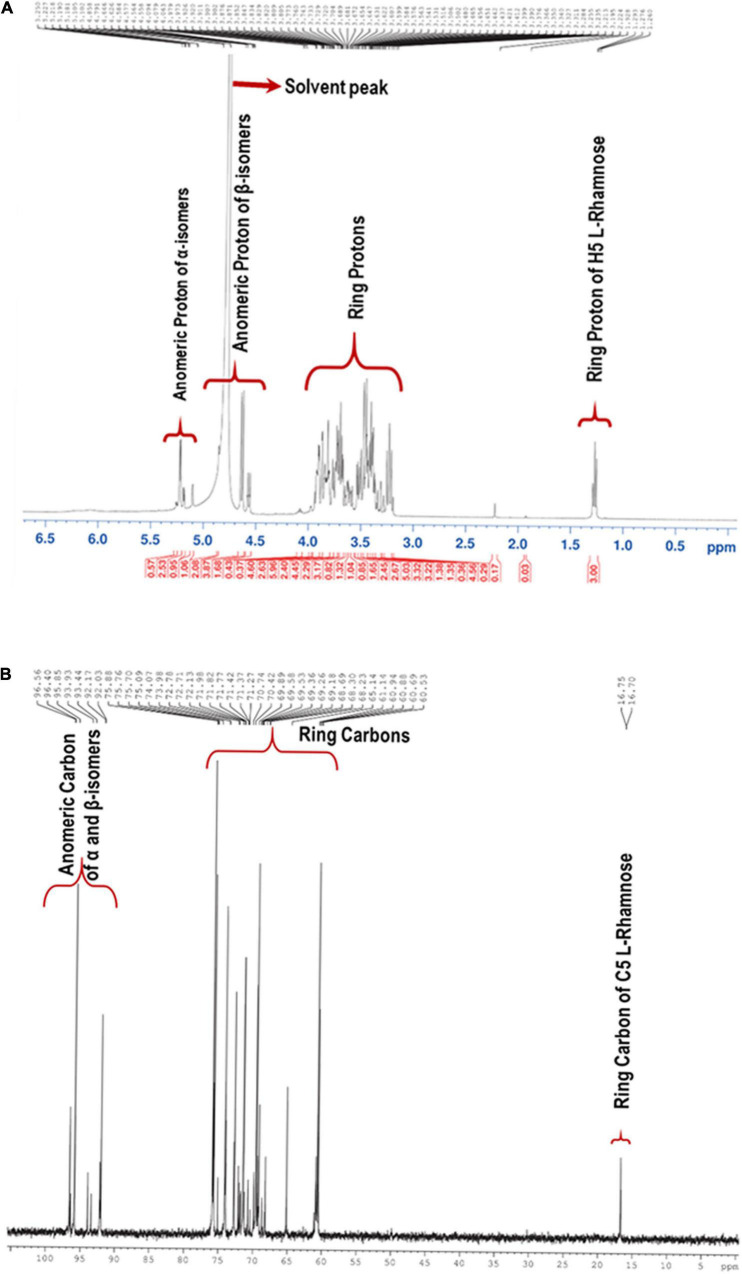
**(A)** Proton (^1^H) and **(B)** Carbon (^13^C) NMR spectra of the EPS purified from *B. albus* DM-15.

NMR spectra of the partially hydrolyzed EPS were recorded with a 400 MHz NMR spectrometer. Figure 3A shows the ^1^H NMR spectrum of the EPS, and most of the signals were clustered between δ 3.23 and δ 5.10. The presence of distinct signals at δ 4.85, δ 4.64, δ 4.57, and δ 4.55 correspond to anomeric protons with *J* = 7–8 Hz demonstrates that four β–isomer sugar units were present in the mixture. The remaining four sets of signals for anomeric protons at δ 5.26, δ 5.22, δ 5.18, and δ 5.10 with *J* = 34 Hz implies that α-anomers were also present. Since the EPS was subjected to hydrolysis using trifluoroacetic acid, the free sugar units adopt α and β forms. The solvent residual peak (deuterium oxide) was obtained at δ 4.79 ppm and one of the anomeric protons, thus confirmed by 2D ^1^H-^13^C HSQC NMR. Similarly, in the ^13^C-NMR, the anomeric carbons appeared at anomeric carbon regions δ 96.61, 95.68, 92.20, 93.97, 93.46, 92.24 92.03 ppm, which confirms the carbohydrate polymer (Figure 3B).

**FIGURE 4:**
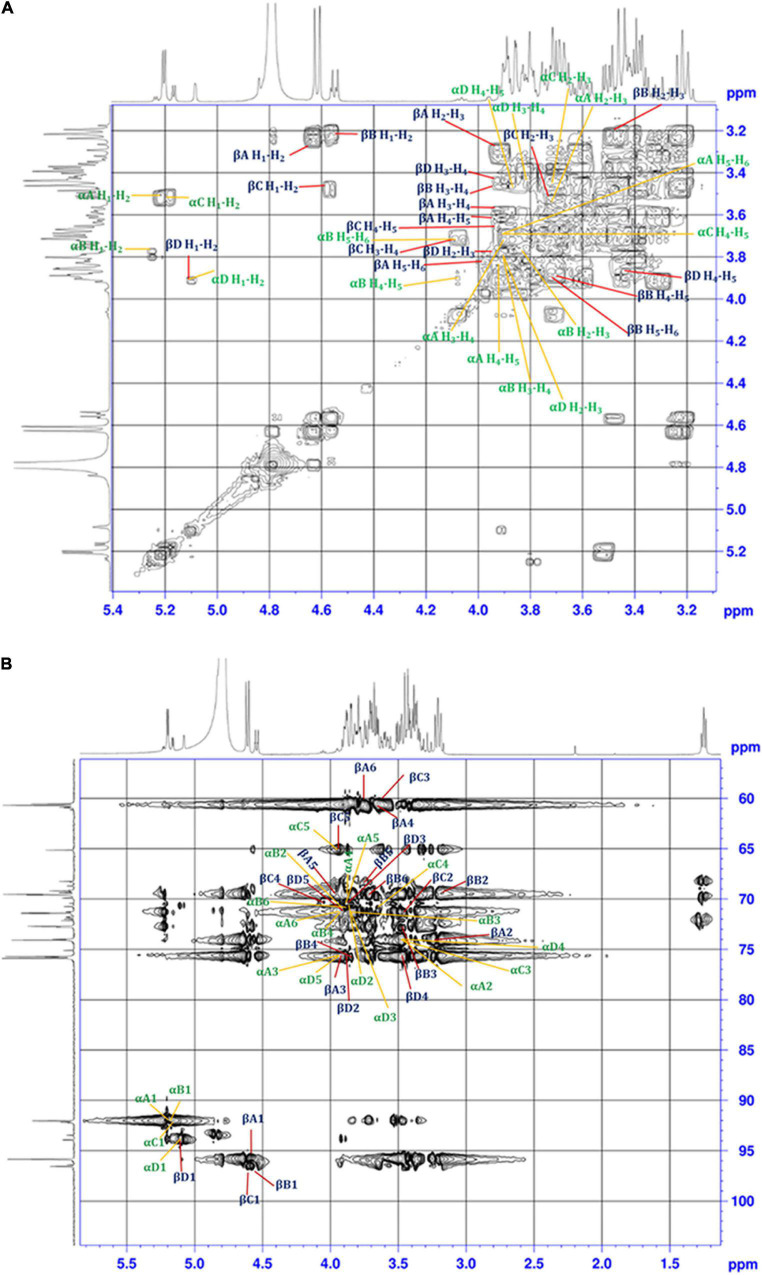
**(A)** ^1^H-^1^H COSY, and **(B)** ^1^H-^13^C HSQC 2D-NMR spectra of the EPS purified from *B. albus* DM-15.

The ^1^H-^1^H COSY technique was used to identify the coupled protons of each sugar unit. The cross-correlation sequence from the α and β anomeric protons with other protons in the ^1^H-^1^H COSY spectrum confirmed D-Glucose and D-Galactose D-Xylose, and L-Rhamnose, which were assigned as residue A, B, C, and D, respectively (Figure 4A). The set of α/β signals at δ 5.22/4.64 (H_1_), 3.50/3.23 (H_2_), 3.77/3.92 (H_3_), 3.91/3.62 (H_4_), 3.97/3.99 (H_5_), 3.72/3.82 (H_6_) were assigned to D-Glucose. The signals at δ 5.26/4.55, 3.79/3.23, 3.82/3.48, 3.89/3.92, 4.08/3.70, and 3.71/3.74 were assigned to α/β form of D-Galactose. The presence of δ 3.80/3.87 (H_5_) corresponds to methylene protons confirms the existence of α and the β state of D-Xylose in the solution. Furthermore, cross-correlation sequence in the ^1^H-^1^H COSY spectrum, the signal at δ 5.18/4.57 with signals at δ 3.52/3.50, 3.63/3.68, 3.63/4.07, 3.91/3.87 were assigned to α/β form of D-Xylose. The exclusive signal at δ 1.28 of methyl group and cross-correlation sequence with anomeric proton leads to the assignment of signals at δ 5.10/4.85 (H_1_), 3.91/3.91 (H_2_), 3.80/3.80 (H_3_), 3.48/3.43 (H_4_), 3.88/3.85 (H_5_), 1.28/1.28 (H_6_) to α/β form of L-Rhamnose. Cross peaks of coupled protons in sugar residues H_5_-H_6_ coupling of β-L-Rhamnose were not shown in Figure 4A. A similar proton NMR signal was obtained at δ 1.28 for Rhamnose residue (Sun et al., 2020).

Single bond correlations between ^1^H and ^13^C were established using the HSQC technique to assign the ^13^C signals of the compounds and were shown in Table 1 and Figure 4B. The anomeric α and β carbons of D-Glucose, D-Galactose, D-Xylose and L-Rhamnose were assigned to δ 92.02/95.94, 92.02/96.59,92.11/96.45, and 93.94/92.05 respectively. The other carbon signals were assigned and represented (Figures 3A,B and Table 1). Overall, the NMR findings strongly support the monosaccharide composition obtained from the GC-MS analysis (Supplementary Figure 4).

**TABLE 1:** Chemical shifts (ppm) of ^1^H and ^13^C (in parentheses) NMR signals for the partially hydrolyzed EPS, recorded in D_2_O at 323 K.

Residue	Sugar unit		Chemical shifts (ppm)					
			H_1_/C_1_	H_2_/C_2_	H_3_/C_3_	H_4_/C_4_	H_5_/C_5_	H_6_/C_6_
A	D-Glucose	α	5.22/92.02	3.50/74.24	3.77/71.19	3.91/71.39	3.97/70.45	3.72/71.40
		β	4.64/95.94	3.23/74.22	3.92/75.92	3.62/72.95	3.99/68.77	3.72/60.60
B	D-Galactose	α	5.26/92.02	3.79/70.52	3.82/71.38	3.92/70.54	4.08/70.43	3.71/71.33
		β	4.55/96.59	3.23/69.59	3.48/72.78	3.92/75.79	3.70/70.49	3.74/68.25
C	D-Xylose	α	5.18/92.11	3.52/71.21	3.63/75.97	3.63/71.12	3.91/64.93	–
		β	4.57/96.45	3.50/71.42	3.68/60.64	4.07/70.39	3.87/65.14	–
D	L-Rhamnose	α	5.10/93.94	3.91/70.61	3.80/71.41	3.48/74.15	3.88/74.97	1.28/16.79
		β	4.85/92.05	3.91/75.79	3.80/71.41	3.43/75.89	3.85/72.64	1.28/16.79

### Microscopic Observation

**FIGURE 5:**
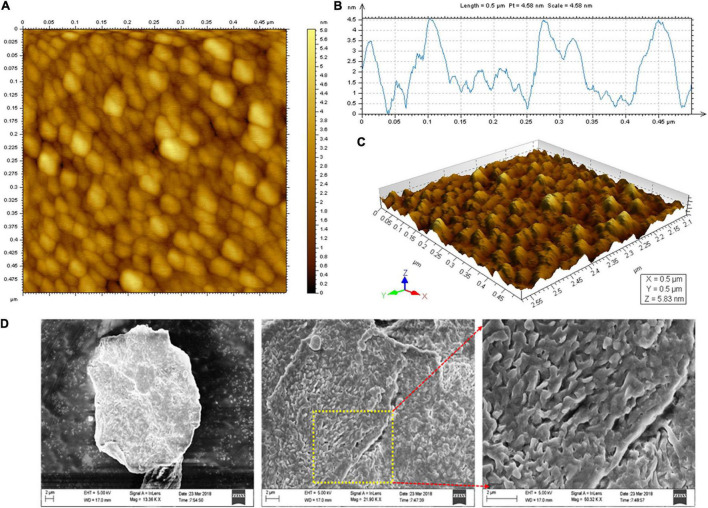
Microscopic analysis of EPS. **(A)** AFM Planar view, **(B)** AFM Transverse profile, and **(C)** Cubic view of AFM images of the EPS, and **(D)** SEM images of the purified EPS.

The spherical lumps with an optimal height of 5.9 nm and fibrous chain-like characteristics were observed under AFM (Figures 5A,B). The 3-D structural view of the AFM images is shown in Figure 5C. The spherical complexes appeared much larger than the chain-like carbohydrates, demonstrating the inter/intramolecular level aggregation in EPS. The spherical, irregular spikes and chain-like structural characteristics of the purified EPS might have fibrous networks in some regions. In contrast, coarse areas were also observed, which indicates the tangled webs in the EPS. EPS from probiotic *B. licheniformis* AG-06 and *L. plantarum* YW11 exhibits similar tangled web-like structural features (Wang et al., 2015; Vinothkanna et al., 2021).

The surface characteristics and microstructure of the EPS were investigated by SEM imaging analysis. The 3-D complexity of EPS was identified and found to be stable. They have predominantly appeared as a tightly porous web-like structure with the irregular coarse surface of stacked flake-like polysaccharides at higher magnification (Figure 5D). EPS derived from *B. licheniformis* PASS26 and *B. licheniformis* AG-06 have exhibited a similar type of porous web-like with polymer matrix structure (Insulkar et al., 2018; Sathishkumar et al., 2021). These permeable web-like molecules are desirable in food industries to increase the porosity and water holding capability and strengthen the materials’ functional properties.

### Flocculation Activity

**FIGURE 6:**
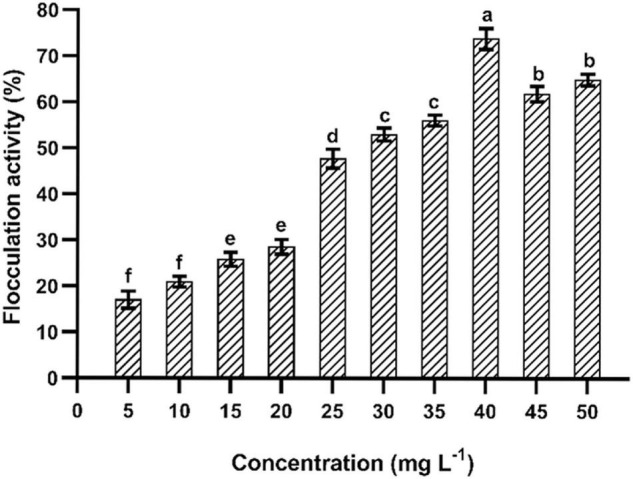
Flocculation of EPS obtained from *B. albus* DM-15. The data were shown as mean ± SD (*n* = 3). Significant differences were determined by Tukey’s HSD test at *p* < 0.05. In addition, values with different letters (a, b, c, d, e, and f) are significantly different.

The purified EPS had shown a significant flocculation activity (Figure 6). The maximum flocculation activity was obtained (73.15 ± 1.25%) at an EPS concentration of 40 mg L^–1^. The flocculation rate was dependent on the optimum dosage, and similar patterns have been found in EPSs isolated from *Pseudomonas* sp. PAMC 28620 and *B. cereus* KMS3-1 (Sathiyanarayanan et al., 2016; Krishnamurthy et al., 2020). It has been strongly believed that the functional groups present in the EPS and molecular weight play a critical role in flocculation activity (Okaiyeto et al., 2016; Nouha et al., 2018). Earlier reports on EPSs purified from *Lysinibacillus fusiformis* KMNTT-10 and *B. cereus* KMS3-1 had shown flocculation activity of 89.66 and 88.35%, respectively (Krishnamurthy et al., 2020; Mathivanan et al., 2021). At present, synthetic/inorganic flocculants are widely used in industries. However, most of them are non-biodegradable, highly susceptible to pH, inefficient in chilled water, and develop an undesirable odor and palatability induce harmful impacts to humans (Okaiyeto et al., 2016). Therefore, naturally extracted flocculants, especially bacterial EPSs, are recommended due to their unique characteristics: innocuous, biodegradability, biocompatibility (effective in pH, cold and warm water), and better quality flocculation efficacy (Okaiyeto et al., 2016). The EPS purified from *B. albus* DM-15 had shown a significant flocculation activity, which can be further exploited for commercial purposes after an intensive screening with other commercially available flocculants.

### Emulsification Activity

**FIGURE 7:**
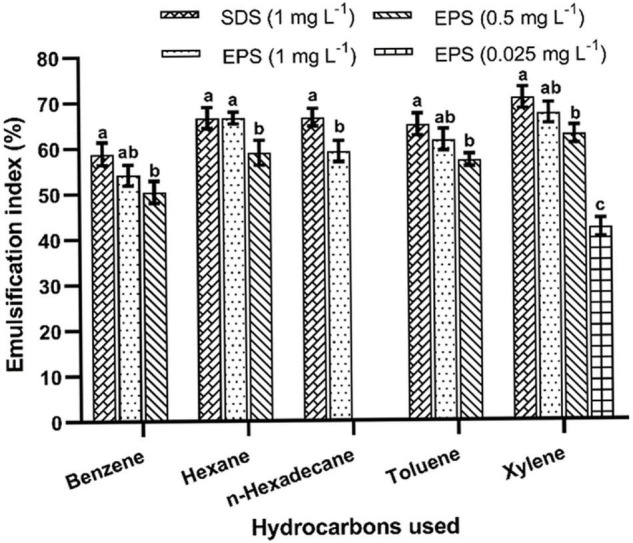
Emulsification activities of EPS obtained from *B. albus* DM-15. The data were shown as mean ± SD (*n* = 3). Significant differences were determined by Tukey’s HSD test at *p* < 0.05. In addition, values with different letters (a, b and c) are significantly different.

The emulsifying activity was determined using different hydrocarbons (Figure 7). The highest emulsification was recorded at 1% of EPS concentration. The purified EPS (0.025%) had shown a maximum emulsification index (67.04 ± 1.75%) with xylene followed by hexane, toluene, n-hexadecane, and benzene after 24 h of incubation. Similar kinds of the emulsifying potential of the EPS have been reported (1% dose) from *B. tequilensis* (63.64%) and *B. pumilus* UW-02 (68%) (Chowdhury et al., 2011; Abid et al., 2019). The emulsification potential of the EPS is a unique characteristic, and the EPS obtained from *B. albus* DM-15 had shown a considerable emulsification activity, demonstrating the promising nature of the EPS with industrial implications.

### Antioxidant Activities

**FIGURE 8:**
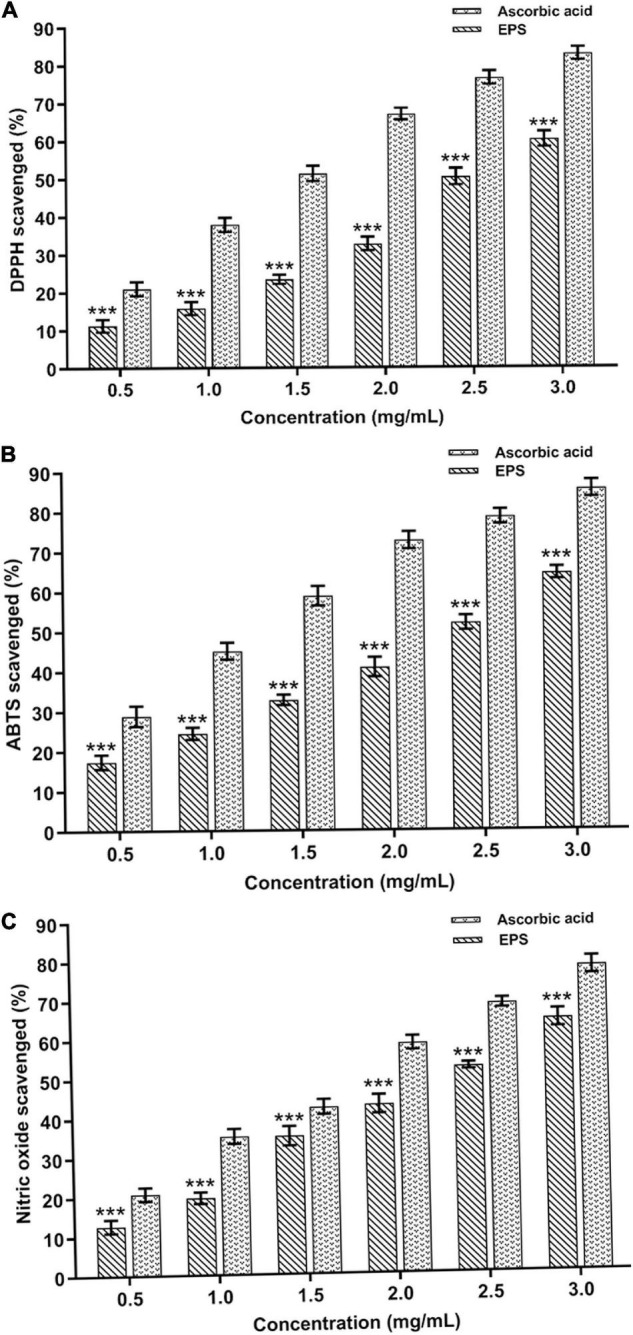
*In vitro* antioxidant potential of the EPS extracted from *B. albus* DM-15. **(A)** DPPH, **(B)** ABTS, and **(C)** Nitric oxide radical scavenging activities of the EPS. The data were shown as mean ± SD (*n* = 3). The significant levels are expressed as *p < 0.05, **p < 0.01, and ***p < 0.001.

The purified EPS and reference standard (L-ascorbic acid) had shown their potential to scavenge DPPH radicals at various doses (0.5–3.0 mg mL^–1^) (Figure 8A). About 59.91 ± 1.44% quenching activity was observed at 3.0 mg mL^–1^ of EPS concentration, which was significantly (*p* < 0.05) lesser than the reference standard (82.76 ± 1.71%). The DPPH free radical quenching activity of EPS has increased in a dose-dependent way, wherein the concentration of the EPS is directly proportional to the antioxidant activity. DPPH activity was comparatively higher than the previously reported EPSs isolated from *Paenibacillus polymyxa* EJS-3 (45.4%) and *B. aerophilus* rk1 (57.6%), wherein 4 mg mL^–1^ of the EPS was used for the DPPH assay (Liu et al., 2010; Gangalla et al., 2021). Furthermore, EPS exhibits the ABTS quenching activity of about 64.24 ± 1.53% at the maximum EPS concentration of 3.0 mg mL^–1^, which is considerably lesser than L-ascorbic acid (85.99 ± 1.65%) (Figure 8B). The ABTS radical scavenging activity trend was similar to DPPH as the activity escalated on increasing EPS concentration. Previous studies also suggest that the EPS isolated from the *B. velezensis* SN-1 EPS (63.3%) and *Pediococcus pentosaceus* M41 (48.9%) had shown a lower ABTS radical scavenging at the maximum dose of 8 and 10 mg mL^–1^, respectively (Ayyash et al., 2020; Cao et al., 2020). The purified EPS had shown an excellent nitric acid quenching activity in a dose-dependent manner with the highest activity of 63.78 ± 1.75% at 3.0 mg mL^–1^ of EPS concentration (Figure 8C). The results obtained from this study was highly corroborated with the EPS purified from the *Lysinibacillus fusiformis* KMNTT-10 EPS, where 3 mg mL^–1^ was used for nitric oxide (NO) scavenging activity (Mathivanan et al., 2021). Therefore, purified EPS might potentially combat the generation of nitric oxide radicals and manage the detrimental effects of nitric oxide. Our earlier report also showed many antioxidant activities, including DPPH (58.17 ± 0.054%), ABTS (70.47 ± 0.854%), and NO (58.92 ± 0.744%), wherein cell-free supernatant of the *B. albus* DM-15 was used for the screening of the antioxidant activities (Vinothkanna and Sekar, 2019). The results obtained from this study were close to our previous study since the EPS was purified from the same strain DM-15. Sulfated EPS purified from *Bacillus megaterium* PFY-147 had shown a significant free radical scavenging activity since the sulfate group in the EPS possibly enhanced the antioxidant and probiotic activities (Pei et al., 2020). In this study, the EPS obtained from *B. albus* DM-15 had shown to have sulfate molecules (119.71 mg/g), possibly strengthening anticancer, antioxidant and probiotic properties. The functional groups present in the EPS, especially the hydroxyl group (-OH), may neutralize the hydroxyl free radicals, thus increasing the quenching ability (Zhao et al., 2018). In addition, the FT-IR analysis also validates the presence of different functional groups (carboxylic acids, ether, alkynes, carbonyl) that may play a key role in therapeutic applications of the EPS (Xu et al., 2011; Zhao et al., 2018; Farinazzo et al., 2020). Overall, antioxidant activities of the purified EPS suggest that this EPS candidate can be exploited further in detail to fully explore the potential of EPSs from the probiotic bacteria isolated from the Indian Ayurvedic fermented medicines.

### Anticancer Potential of the Exopolysaccharide

In this study, the cytotoxic effect of the EPS against A549 cells was observed with an IC_50_ value of 20 ± 0.97 μg mL^–1^ (Supplementary Figure 5). The cytotoxic activity of the EPS is directly proportional to the concentration of the EPS. Our results also substantiate with the EPSs purified from *B. altitudinis* MSH2014 and *B. licheniformis* AG-06, which inhibits the proliferation of the A549 cancer cells (Mohamed et al., 2018; Vinothkanna et al., 2021). Antiproliferative or cytotoxic activity is quite common in sulfated EPS. For example, EPS from *Anoxybacillus gonensis* YK25 showed considerable anticancer activity against lung cancer cells (Karadayi et al., 2021). The EPS used in this study also possess the sulfate content, which might be responsible for the cytotoxic activity.

**FIGURE 9:**
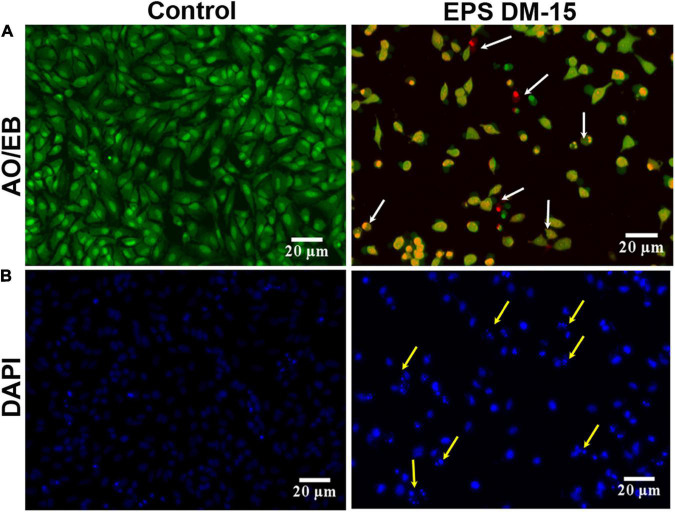
Anticancer activity of the EPS obtained probiotic *B. albus* DM-15 against lung cancer cell line A549. Cytotoxic effect of the EPS against A549 cells after 24 h exposure. **(A)** AO/EB staining with the IC_50_ concentration of EPS. Green-colored are intact live cells, and orange-colored (white arrow) are dead exhibiting apoptotic morphology. **(B)** DAPI staining with the IC_50_ concentration of EPS revealed healthy and damaged cells (yellow arrow).

In addition, the IC_50_ dose of the EPS was used to evaluate the AO/DAPI staining of A549 cancer cells (Figure 9A). The intact viable cells in the control group appeared as fluorescent green. At the same time, EPS-treated damaged cells were orange, distinctly demonstrating apoptotic characteristics such as chromatin condensation and cell shrinkages (early apoptosis) and cell fragmentation (late apoptosis). Also, very few cellular necroses were observed in EPS-treated cells. DAPI staining of cell nuclei treated with EPS had revealed the apoptotic and a few necrotic characters in damaged cells (Figure 9B). Other cytological and apoptotic modifications, including membrane blebbing and the development of apoptotic bodies (condensed dots), were found in EPS treated cancer cells. Furthermore, a maximum amount of damage was found in the EPS treated cells compared to control (healthy cells) (Supplementary Figure 6). It demonstrates that the EPS from *B. albus* DM-15 is a promising candidate against the A549 lung cancer line. Further intense studies on this EPS molecule will possibly open the doors to formulate potential EPS-based dietary supplements and natural drugs for cancer prevention and treatment, particularly lung cancer.

## Conclusion

This study emphasizes the EPS production, purification, and characterization from *B*. *albus* DM-15 isolated from the Indian ayurvedic fermented medicine *Dasamoolarishta.* GC-MS and NMR analysis confirm the heteropolymeric nature of the purified EPS composed of glucose, galactose, xylose, and rhamnose. The purified EPS had shown a molecular weight of about 240 kDa. X-ray diffraction (XRD) analysis confirmed the non-crystalline amorphous nature of the carbohydrate polymer. AFM and SEM analyses also demonstrate the fibrous and web-like complexity with an irregular coarse surface of stacked flake-like porous nature of the EPS. Furthermore, the EPS has exhibited significant flocculation and emulsification properties. In addition, the EPS had shown potential antioxidant and anticancer activities. Therefore, EPS obtained from probiotic *B. albus* DM-15 can be further studied for nutraceutical and pharmaceutical applications.

## Data Availability Statement

The original contributions presented in the study are included in the article/Supplementary Material, further inquiries can be directed to the corresponding author/s.

## Author Contributions

AV: conceptualization, methodology, formal analysis, validation, visualization, writing, and original draft. GS: validation, writing—original draft, and writing—review and editing. AR: visualization, review, and editing. KM: formal analysis and visualization. KSa, KSu, and PK: formal analysis. YM: project administration, validation, visualization, and writing—review and editing. SS: conceptualization, supervision, project administration, funding acquisition, and writing—review and editing. All authors contributed to the article and approved the submitted version.

## Conflict of Interest

The authors declare that the research was conducted in the absence of any commercial or financial relationships that could be construed as a potential conflict of interest.

## Publisher’s Note

All claims expressed in this article are solely those of the authors and do not necessarily represent those of their affiliated organizations, or those of the publisher, the editors and the reviewers. Any product that may be evaluated in this article, or claim that may be made by its manufacturer, is not guaranteed or endorsed by the publisher.
