# Supplementary material for: Exopolysaccharide Produced by Probiotic Bacillus albus DM-15 Isolated From Ayurvedic Fermented Dasamoolarishta: Characterization, Antioxidant, and Anticancer Activities
Source: Front Microbiol. 2022 Mar 3;13:832109. doi: 10.3389/fmicb.2022.832109 (PMC8927020; doi:10.3389/fmicb.2022.832109)
Supplement: Supplementary file 1 [file Data_Sheet_1.PDF]

Exopolysaccharide produced by probiotic *Bacillus albus* DM-15 isolated from Ayurvedic fermented *Dasamoolarishta*: Characterization, antioxidant and anticancer activities

Annadurai Vinothkanna[a,b], Ganesan Sathiyanarayanan[c], Amit Kumar Rai[d], Krishnamurthy Mathivannan[e], Kandasamy Saravanan[f], Kumaresan Sudharsan[g], Palanisamy Kalimuthu[g], Yongkun Ma[a,*], Soundarapandian Sekar[b,*]

[a] *School of Food and Biological Engineering, Jiangsu University, 301 Xuefu Road, Zhenjiang 212013, PR China.*

[b] *Department of Biotechnology, Bharathidasan University, Tiruchirappalli, 620 024, Tamil Nadu, India.*

[c] *Rue Marie-de-Nemours 3, 2000 Neuchâtel, Switzerland.*

[d] *Institute of Bioresources and Sustainable Development, Regional Centre, Tadong 737102, Gangtok, Sikkim, India*

[e] *School of Minerals Processing and Bioengineering, Central South University, Changsha, Hunan 410083, PR China*

[f] *Department of Biochemistry, Bharathidasan University, Tiruchirappalli, 620 024, Tamil Nadu, India.*

[g] *Department of Chemistry, The Gandhigram Rural Institute (Deemed to be University), Gandhigram, Dindigul 624302, Tamil Nadu, India*

*** Corresponding authors:**

Dr. Yongkun Ma, School of Food and Biological Engineering, Jiangsu University, Zhenjiang 212013, PR China. E-mail address: mayongkun@ujs.edu.cn

Dr. Soundarapandian Sekar, Department of Biotechnology, Bharathidasan University, Tiruchirappalli, 620 024, Tamil Nadu, India. E-mail address: sekarbiotech@yahoo.com

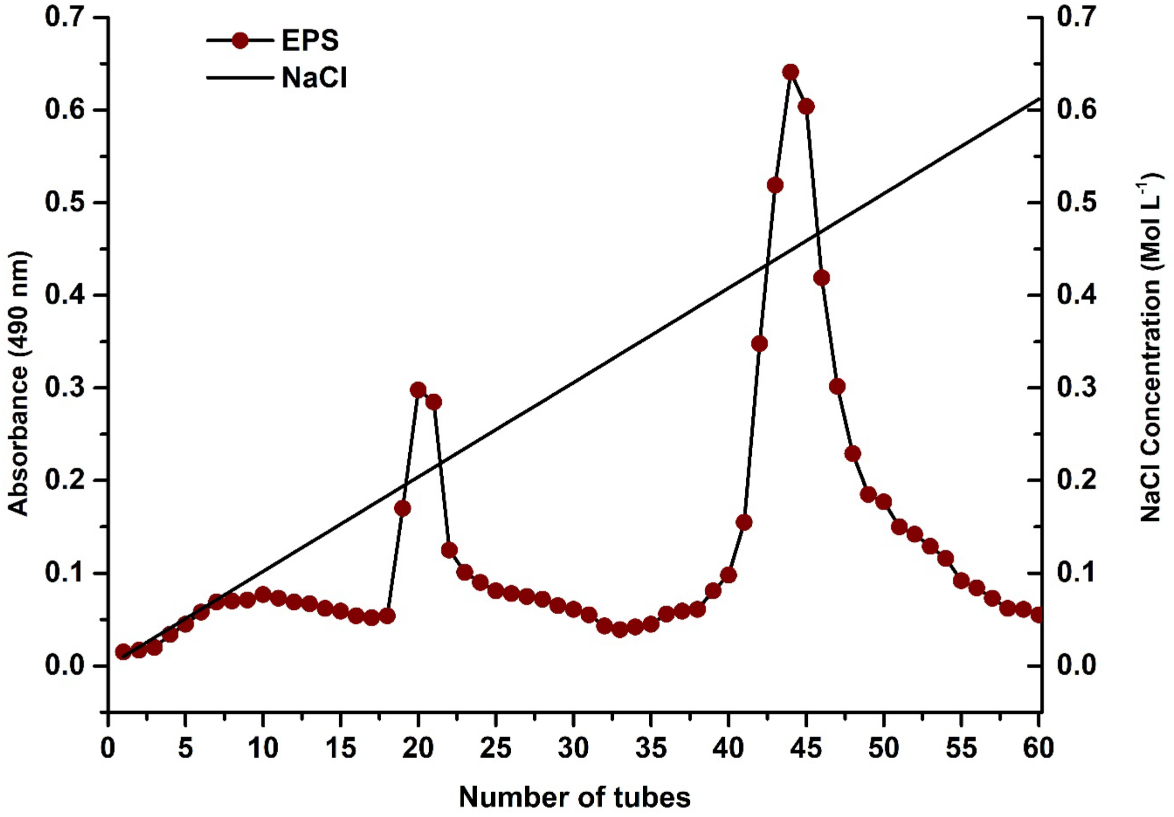

Figure S1. Anion-exchange chromatography for the purification of the EPS extracted from the probiotic *Bacillus albus* DM-15. NaCl was used as the eluent.

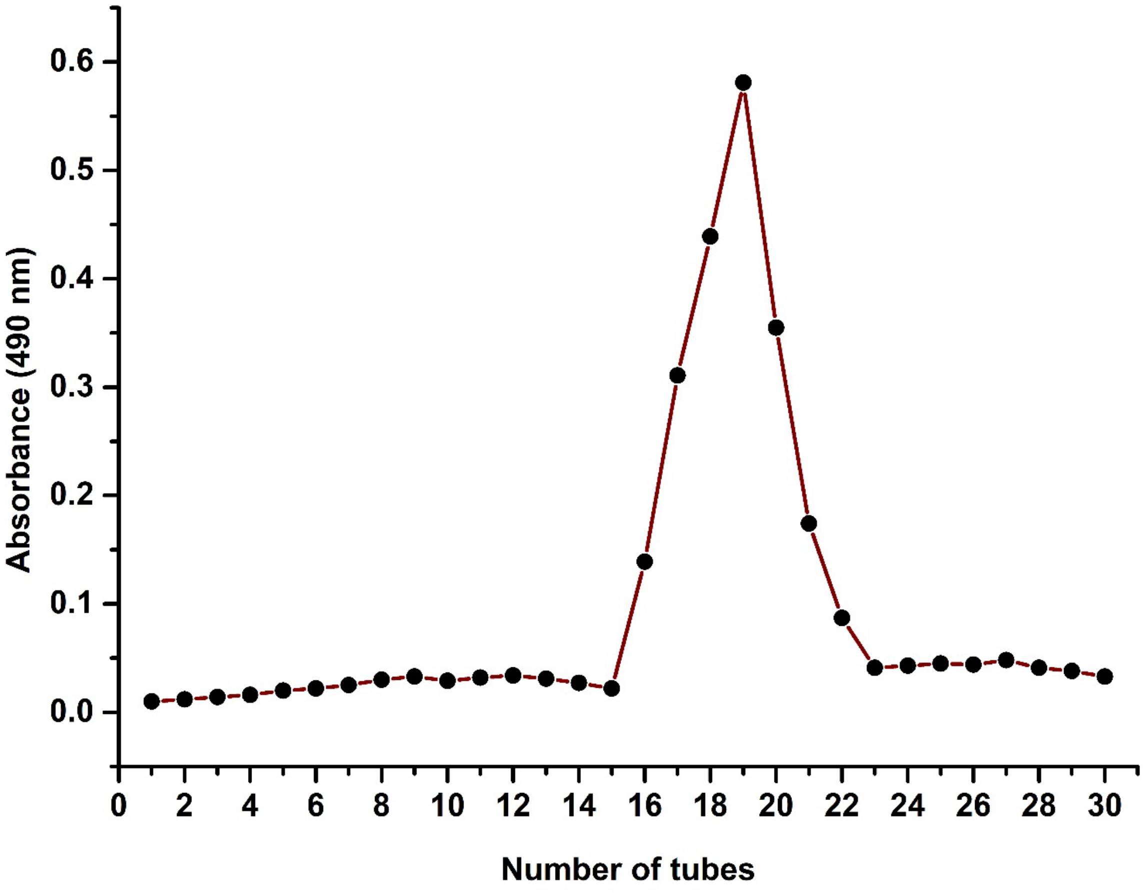

Figure S2. Gel-filtration chromatography for the purification of the EPS extracted from the probiotic *Bacillus albus* DM-15. Deionized water was used as the eluent.

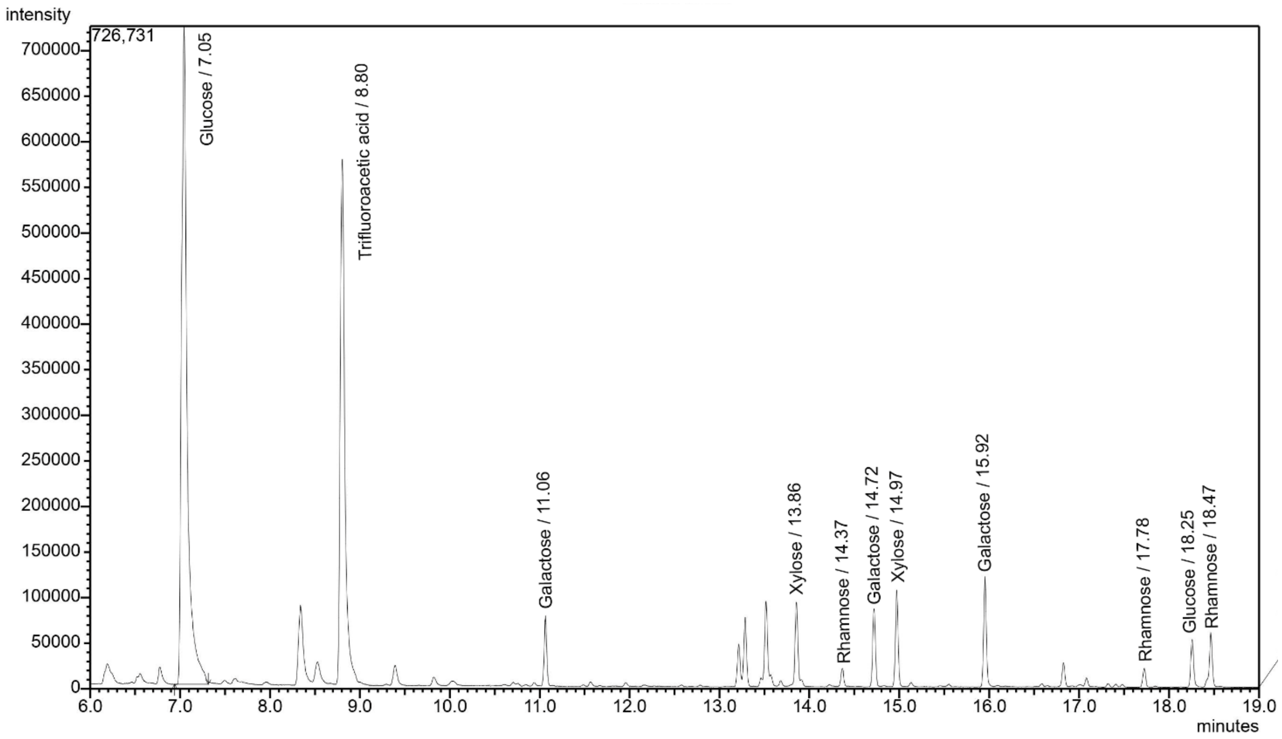

Figure S3. GC-MS spectrum of glycosyl composition of the EPS from probiotic *B. albus* DM-15

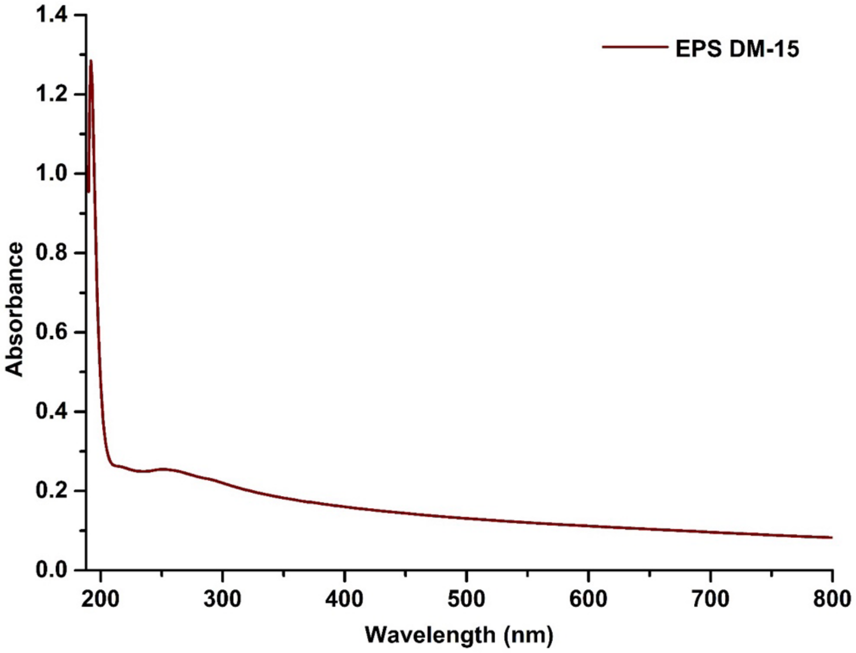

Figure S4. UV-Visible spectrum of the EPS purified from *Bacillus albus* DM-15

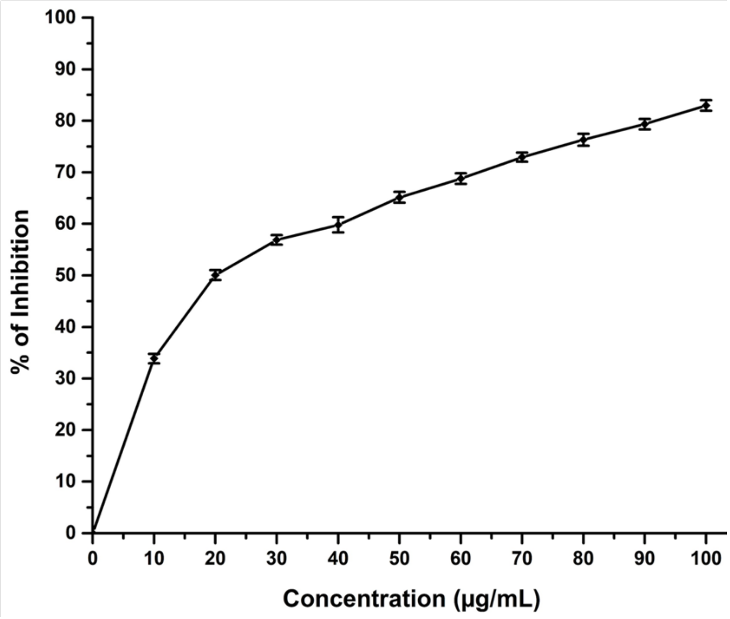

Figure S5. Cytotoxic effect of EPS against A549 lung cancer cells after exposure for 24 h.

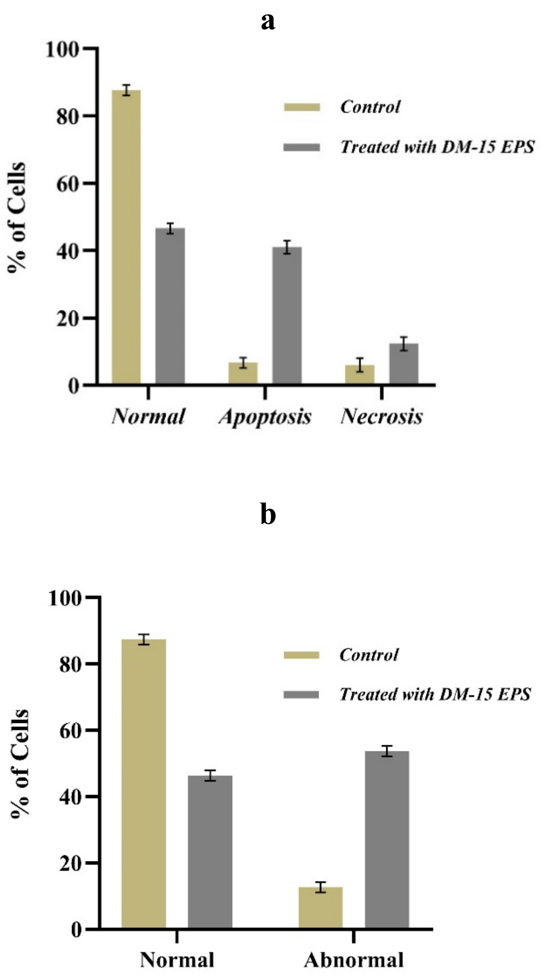

Figure S6. Total cell counts in (a) AO/EB and (b) DAPI staining fluorescent analysis.

Table S1. Glycosyl composition of the EPS from *B. albus* DM-15.

**Monosaccharide units**	**Retention time (min)**	**Area**	**Amount[a] (mol %)**
Glucose	7.05, 18.25	2974028	71.33
Galactose	11.06, 14.72, 15.92	564859	13.55
Xylose	13.86, 14.97	391097	9.38
Rhamnose	14.37, 17.78, 18.47	239611	5.75
